# Astroglia in Thick Tissue with Super Resolution and Cellular Reconstruction

**DOI:** 10.1371/journal.pone.0160391

**Published:** 2016-08-05

**Authors:** Sean J. Miller, Jeffrey D. Rothstein

**Affiliations:** 1 Department of Neurology, Johns Hopkins University School of Medicine, Baltimore, MD, United States of America; 2 The Brain Science Institute at Johns Hopkins University, Baltimore, MD, United States of America; 3 Cellular and Molecular Medicine, Johns Hopkins University School of Medicine, Baltimore, MD, United States of America; Albany Medical College, UNITED STATES

## Abstract

We utilized the recently published method of passive CLARITY to explore brain astrocytes for the first time with our optimized method. Astrocytes are the fundamental cells in the brain that act to maintain the synaptic activity of neurons, support metabolism of all neurons, and communicate through extensive networks throughout the CNS. They are the defining cell that differentiates lower organisms from humans. From a disease vantage point they are the principal cause of brain tumors and the propagator of neurodegenerative diseases like amyotrophic lateral sclerosis. New methods to study these cells is paramount. Our modified use of CLARITY provides a new way to study these brain cells. To reduce cost, speed up tissue clearing process, reduce human handling error, and to retrieve quantifiable data from single confocal and pseudo-super resolution microscopy we modified and optimized the original protocol.

## Introduction

The most abundant cell type in the adult brain are astrocytes, and yet the multiplicity of functions that astroglial serve is poorly understood[1]. For decades’ scientists have appreciated the fundamental support that astroglia provide to neurons, including neurotrophic support, ion and neurotransmitter homeostasis, synapse pruning, and perineuronal nets[2–4]. Astrocytes are elaborate cells, that intertwine with all cell types of the CNS and interact with up to 100,000 neuronal synapses per astrocyte in rodent and up to 2 million synapses in humans[5]. However, the roles of different astroglia subpopulations and their involvement with the local cellular environment *in vivo* still remains largely unexplored in both health and disease, leaving large gaps in our understanding[1].

In various disorders, astrocyte dysfunction can play a substantial role in the disease progression[6, 7]. In Huntington’s disease, potassium ion imbalance in the striatum induces neuronal hyperexcitability that eventually contributes to neuronal death[8]. Restoring the levels of a potassium channel on the striatal astroglia, diminishes the extracellular potassium dyshomeostasis and reduces Huntington’s disease progression in animal models[8]. In other disorders, such as amyotrophic lateral sclerosis, the astrocyte glutamate transporter, Glt1, is significantly downregulated leading to excess extracellular glutamate and glutamate-induced excitotoxicity to motor neurons[9]. Lastly, astrocyte-like glia of the gastrointestinal tract have been shown to exacerbate the inflammation and progression of disorders such as Crohn’s disease and Ulcerative Colitis[10].

In aggregate, these findings exemplify the influence of astroglia on the local environment in the CNS and enteric system. However, one major challenge to studying astroglia has been the imaging of astroglia in thick tissue with high resolution microscopy to be able to fully appreciate and understand their local and regional biology[11]. Additionally, murine astroglia generally span over 100uM or 23,000um^3^, making observation of their entire cellular structure more challenging[5]. Historically, imaging restrictions were confined to the micron thickness of the histological section or the working distance of the imaging objective which limits the ability to image deep in tissue. Furthermore, prior tissue mounting is almost always an irreversible process where the tissue cannot be re-stained but is rather permanently damaged and mounted. With the recent advances in multiphoton, increased working distance objectives, and tissue clearing methods like CLARITY, it is now possible to image cells deep in tissue in their native biological environment[12]. In our study, we imaged astroglia for the first time in a low-cost optimized CLARITY handling system, with multi- and single-photon confocal microscopy, and at super resolution to improve the imaging techniques currently used to study astroglia. Furthermore, we demonstrate the ability to count and morphologically reconstruct astroglial processes in thick tissue that is made practical for basic research labs to perform.

## Materials and Methods

### Animals

BAC-GLT1-eGFP and 8.3kb-tdTomato mice were used for all experiments[13]. The care and treatment of animals is in accordance with the NIH Guide for the Care and Use of Laboratory Animals, the Guidelines for the Use of Animals in Neuroscience Research, and the Johns Hopkins University IACUC. The protocol for this study was approved by the Johns Hopkins University Animal Care and Use Committee (Protocol number: MO14M89). Mice were housed at standard temperature (21C) and in light controlled environment with ad libitum access to the food and water. BAC-GLT1-eGFP mice were crossed with 8.3kb-tdTomato mice to generate double transgenic mice. No more than five mice were kept in a cage, in accordance with Johns Hopkins University IACUC. Mice were sacrificed between age of P60-P90. Animals were sacrificed with a lethal intraperitoneal injection of ketamine xylazine at 100mg per kg, which is consistent with the recommendations of the Panel on Euthanasia of the American Veterinary Medical Association.

### Passive Clarity with Optical Wells

Passive CLARITY was performed as previously described[12]. Briefly, post fixation tissue was placed into A4P0 (Sigma, A9099; Wako, VA-044) solution at 4C overnight, the following day samples were degassed and filled with inert gas for 2–3 minutes per sample at room temperature, and placed into a 37C water bath will sealed cap for 2–3 hours for hybridization. Post hybridization samples were washed with 0.1M PBS. Brains were then serial sectioned at a thickness that corresponded to the desired-objectives working distance and with corresponding sections placed into optical wells (in vitro scientific, P24-1.5H-N). Optical wells with samples were incubated on a 4C shaker with 8% SDS (Sigma, C3771) in 0.1M PBS for 2–4 days. Samples were then washed in the same optical wells over one day at room temperature with 1X PBS. Next antibody staining (i.e. GFAP, ALDH1L1) would take place or for genetically encoded fluorescent reporters (e.g. eGFP, tdTomato) the samples were immediately bathed with histodenz (Sigma, D2158) solution for 1–3 days until transparent. Prior to imaging, histodenz was removed and replaced with fresh imaging histodenz. After imaging, histodenz was removed and tissue was stored in 1X PBS with 0.1% Triton-X-100 and 0.1% sodium azide until next imaging or staining session. To reduce handling, samples are never removed from the optical wells.

### Single and Multi-Photon Microscopy

Optical well plates with cleared tissue were placed into a microscopy plate holder. Single photon images were obtained on a Zeiss LSM 700 and LSM 800. Multiphoton images were acquired on a Zeiss LSM 710. Pseudo-Super resolution images were acquired on a Zeiss LSM 800 with airy scan. Zeiss Zen 2012 software was used to acquire images. Multiple confocal systems were used to ensure versatility for other users of this methodology. Objectives used were 10x air, 20x air, and 63x oil immersion.

### Software Suite

Bitplane imaging software was used, known as Imaris 8.3, available by purchase of a one-time license (with yearly updates for additional purchase). Furthermore, it is a very user friendly 3D rendering software. Due to its cost, some publically available software are also available including: Vaa 3D for 3D rendering, Image J and Fiji for 2D rendering.

### Automated Cellular Reconstruction

Multiple open source 3D-rendering software exist, both privately and publically available (see methods). For cellular morphological reconstruction, Bitplane Imaris software was used which can be purchased from Bitplane for a one-time license fee. Briefly, images were imported and subjected to surface reconstruction. Surface reconstruction parameters were set to appropriately label all astroglia and their processes. Multiple images were tested for accuracy prior to the parameters being used as an automated reconstruction for astroglia. For cells that had diffuse signal across multiple cells, they were manually disconnected using the Bitplane Imaris software.

### Automated Cell Count

Multiple open source 3D-rending software exist (see methods). For our automated cell counts, Bitplane Imaris software was used. To set parameters, we manually counted astroglia in selected images. Additionally, images were subjected to single Z-slice focus, then using Imaris’s spot detection, cell count parameters were set for size and fluorescence strength of voxels. Parameters were then subjected to multiple image tests between manually counted images and automated cell counts to ensure accuracy of detecting only cells of interest. After validation, parameters were saved and then expedited through entire thick Z-stacks and overall cell count data was obtained for each image.

## Results

### Passive Clarity to Study Astroglia

**Fig 1:**
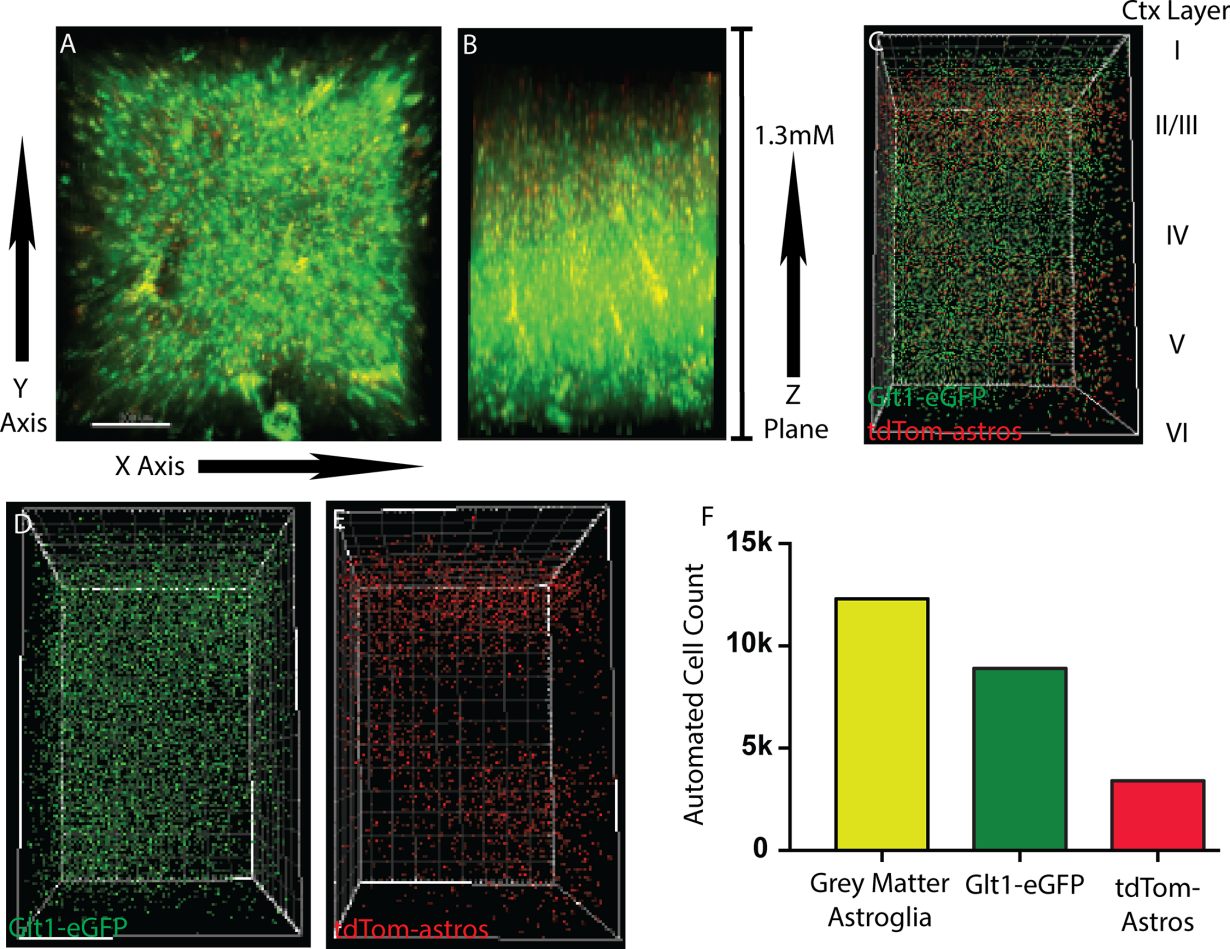
multiphoton imaging of grey matter astroglia. A) Imaging of cleared cortex shows large astroglia populations, b) one multiphoton image of the entire cortex shows astroglia abundance changes, c) each astroglia is automatically represented as one spot based on fluorescence, d) glt1-egfp astroglia are highly abundant throughout all cortical layers, e) an astroglia subtype is only represented and shows cortical layer preference, f) automated cell counting of z-stack counts over 12k cells, consisting of two astroglia populations.

Astrocytes are the most abundant cell type in the brain[1]. It has been reported that there is a 7.7 glia/neuron ratio in the minke whale cortex or 1.4 in the human cerebral cortex[14]. To visualize all grey-matter astroglia we performed passive clearing on the Glt1-eGFP/tdTom-astros mouse, which labels all grey-matter astroglia with eGFP and an astroglia subset with tdTomato/eGFP[12, 13]. Post clearing and sectioning, the entire cortex was placed into the optical well plate and imaged with multiphoton confocal. Starting at the pia and imaging down to the corpus callosum for a total thickness of 1300uM (Fig 1A and 1B; S1 Movie). We then generated a spot using Imaris Spot Detection to represent the localization of each astroglia soma. Overall it appeared a relatively consistent distribution of Glt1-eGFP astroglia throughout the cortical layers, supportive of our unpublished data (Fig 1C; and unpublished data).

Next, we isolated each astroglia population based on their fluorescence emissions (Fig 1D and 1E). There was a clear preference for tdTom-astros in cortical layers II/III and V but no clear separation for Glt1-eGFP astroglia (Fig 1D and 1E). To test our automated cell quantification in very thick tissue, we subjected Bitplane Imaris Spot Detection to count all astroglia and the tdTom-astros subpopulation with parameters previously established (see Fig 1C). We counted over 12,000 astroglia in the timescale of seconds. Importantly, these cell counts were closely correlated to the manual cell counts we performed on thin sliced cortical tissue (Fig 1F; S1A Fig). In comparison to manual cell counting, using automated cell counting of thick sections not only dramatically increased the volume of tissue we could quantify but also sped up this process significantly. Lastly, groups with 3D rendering software such as Imaris can merge sectioned tissue after analyses to generate one large quantifiable image, limiting the need for high computing processors (not shown).

### Astroglia Form Interlocked Networks

**Fig 2:**
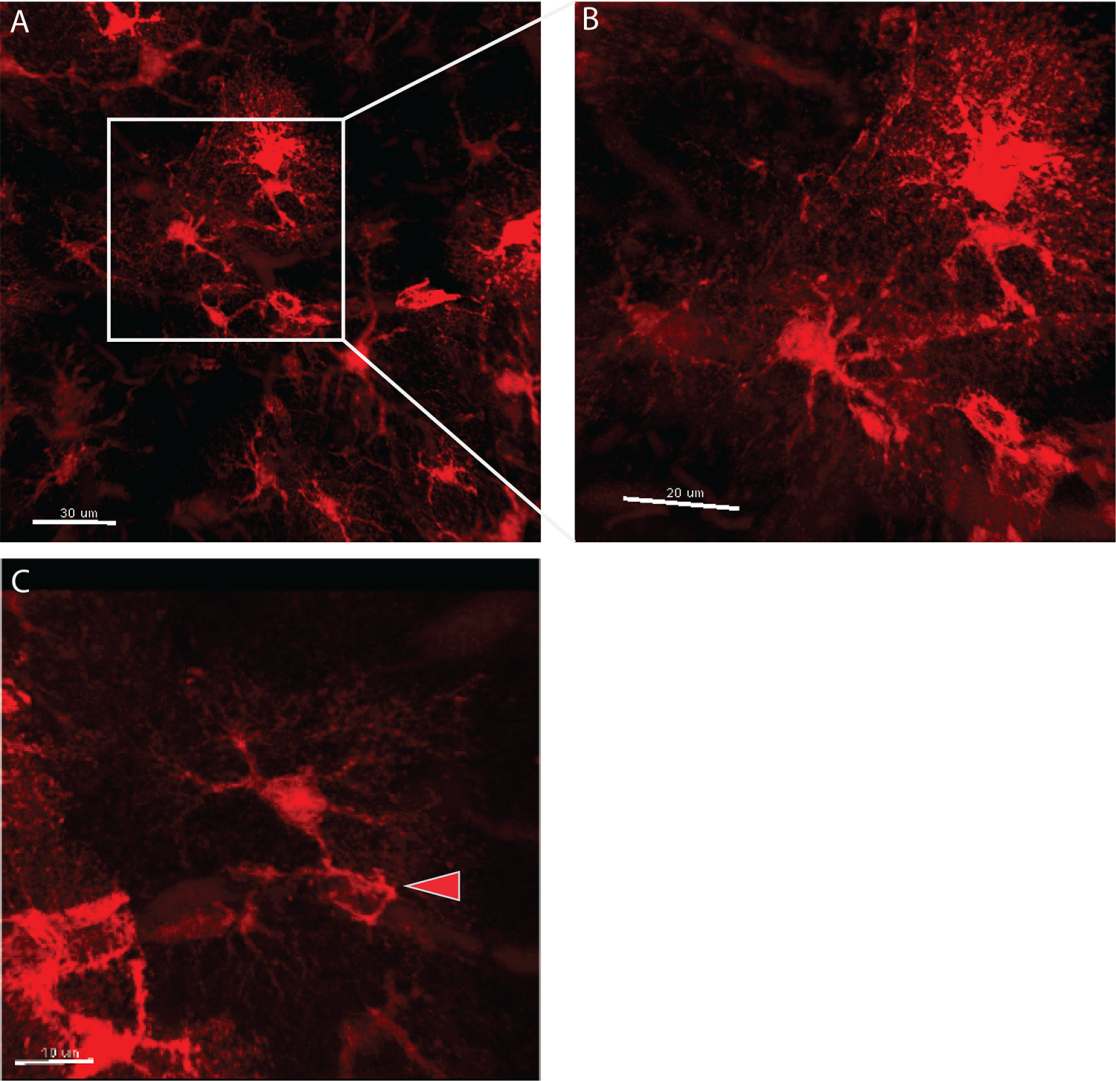
cleared cortex shows highly detailed astroglia processes and interactions. A) astroglia processes interact with other astroglia and cerebral blood vessels, b) computational zoom shows highly branched astroglia processes interacting with other astroglia, c) magnified image of cortical astroglia wrapping a local cerebral blood vessel.

Astroglia communicate through extensive networks and various cell-cell junctions throughout the CNS with high plasticity that changes throughout development, aging, and disease[4]. To examine interconnections between astroglia, tdTom-astros cortical tissue was subjected to passive clarity and imagined with single-photon microscopy. At 20x, we uncovered somewhat more ascertainable morphological details as compared to major GFAP-immunopositive processes, and astroglia-astroglia connections (Fig 2A and 2B; S2 Movie). However, even this method cannot fully visual, in this 3D imaging method, the very fine, peri-synaptic astroglia processes best revealed with electron microscopy. In further examination tdTom-astros formed a clustering-like integration in the cortex, as expected for cortical astroglia where it has been reported that cortical astroglia arise from clonal populations[15] [Fig 2A]. Also, we show a tdTom-astros end foot process projecting to a neighboring cerebral blood vessel (Fig 2C). Lastly these images can be further magnified, analyzed, and reconstructed in the Imaris software suite.

### Astroglia Morphological Reconstruction in Thick Intact Tissue

**Fig 3:**
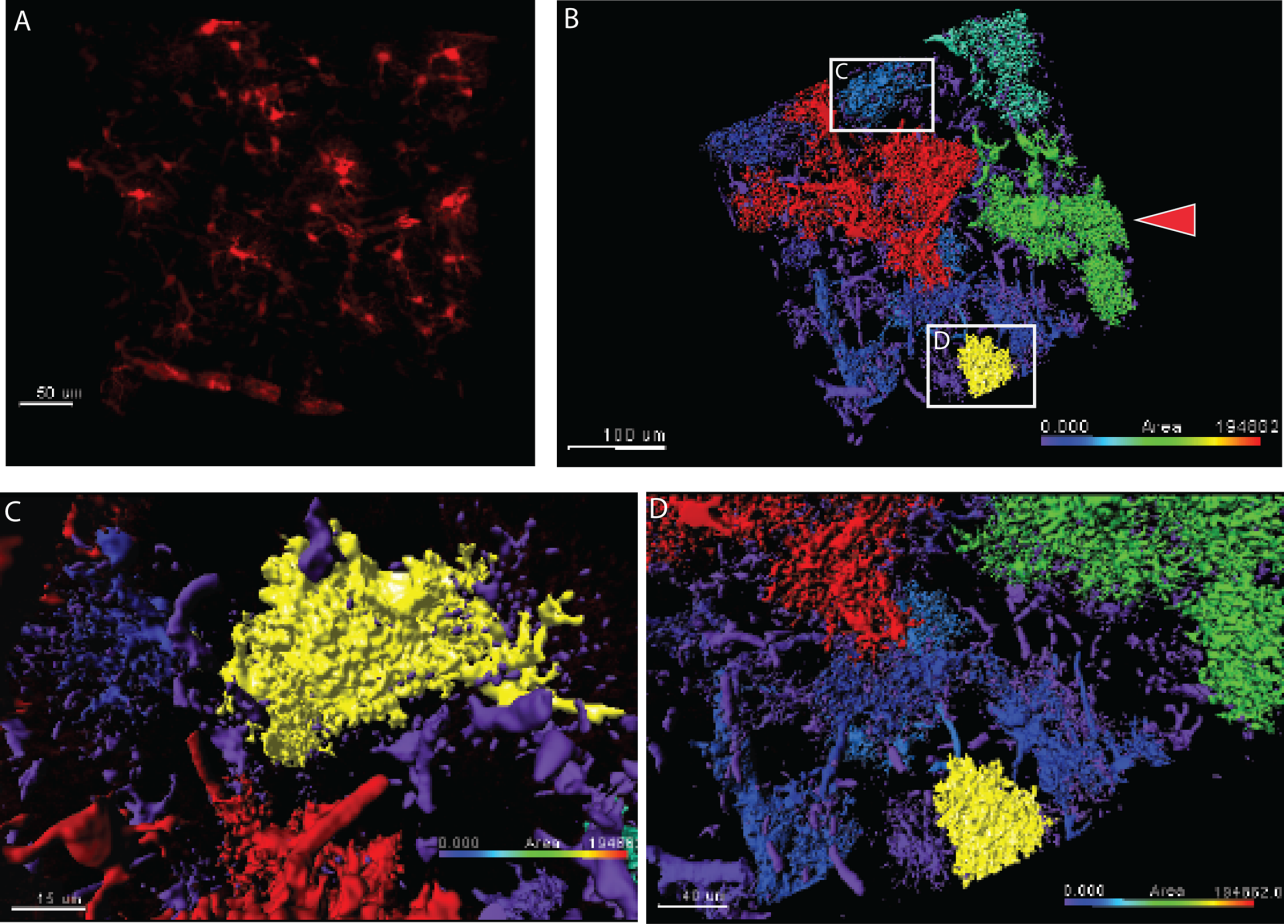
automated morphological reconstruction of cleared astroglia. A) overview of single photon image of grey matter astroglia, b) automated morphological reconstruction of cleared astroglia shows great diversity, c) magnified reconstruction of grey matter astroglia shows highly branched processes, d) magnified reconstruction of grey matter astroglia shows volume diversity amongst astroglia.

Next, we wanted to obtain quantifiable data of the morphology of different astroglia from thick tissue through automated image software-based analytics. It is widely known that astroglia undergo dramatic physical changes during reactivity and CNS changes such as in disease which are important phenotypical changes to quantify and understand. To address this, we generated parameters via Bitplane Imaris software that could be applied to all Z-sections of the imaging data to reconstruct each astroglia based on their volume. Furthermore, using these user-defined parameters, users are capable of processing many Z-stacked images simultaneously from different sections and future samples. In support of prior literature, we found that astroglia vary highly in their cellular volume (Fig 3A, 3B, 3C and 3D; S3 Movie). Astroglia volume difference could be used as an automated tool to study hypertrophy, where astroglia undergo a typical reactive swelling. Lastly we computationally magnified selected astroglia and found dramatic differences in both their process structure and their volume with different values in the micron scale of 2.0e^4^uM^2^ and 4.4e^4^uM^2^, respectively (Fig 3C and 3D). **Note, the Imaris software highlights a user-selected cell as yellow, hence the identified yellow astroglia in Fig 3C and 3D, are in fact the two boxed cells in Fig 3B.** Lastly, morphological reconstruction easily identified clustering astroglia that physically connected and can be manually disconnected for desired analytics (Fig 3B arrow). This reconstruction could be used as a tool to study individual astroglia and their reactivity, or effects on clustered astroglia populations.

### Pseudo Super Resolution Passive Clarity of Astroglia And Their Processes

**Fig 4:**
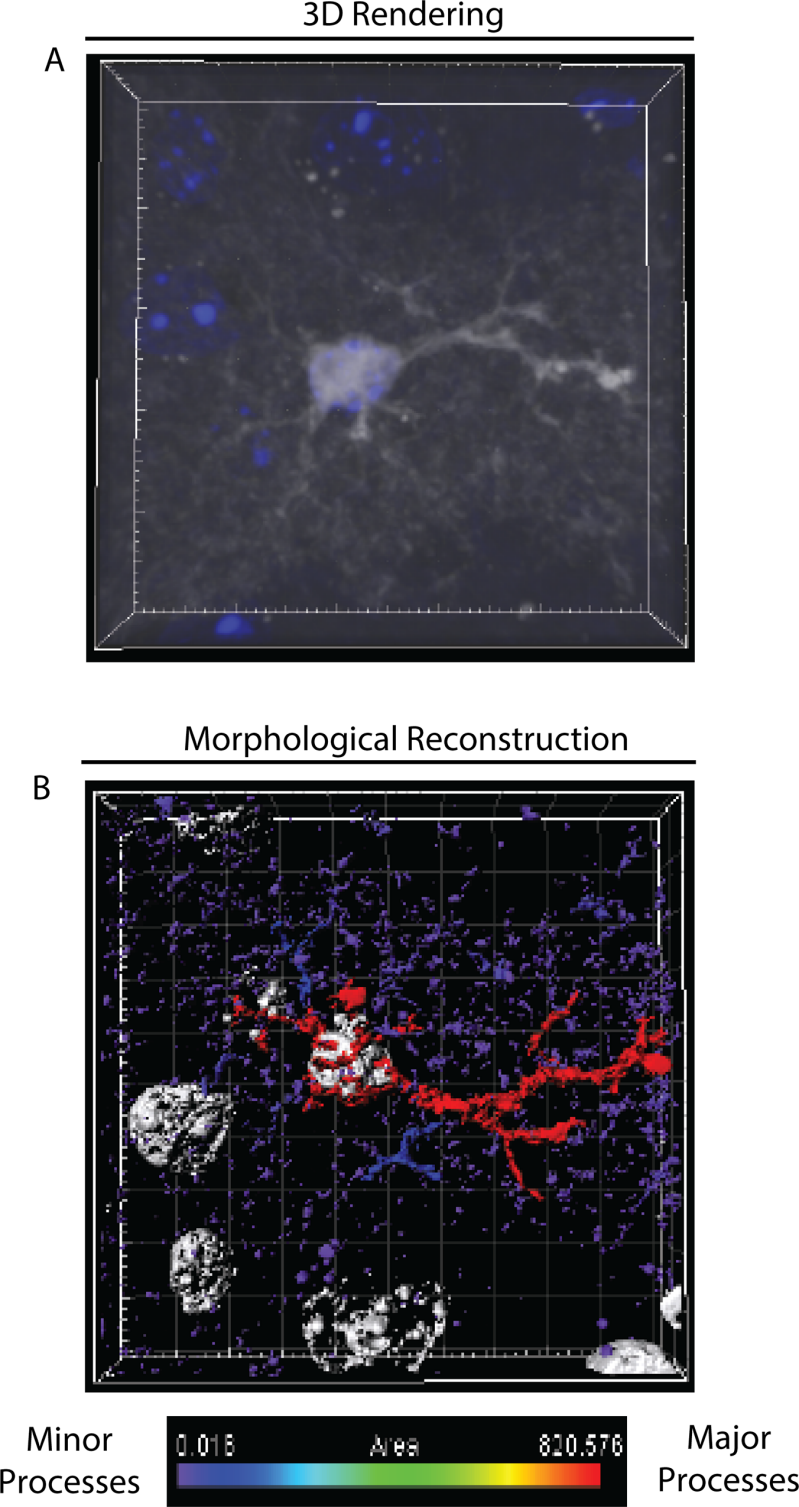
Pseudo super resolution uncovers intimate branching and interactions covering nearby cells. A) 3d-rendering of a single astroglia at super resolution shows overlap with local non-astroglia cells, b) automated morphological reconstruction shows minor and major processes with detailed contextual resolution.

Super resolution significantly increases the resolution of typical confocal microscopy to allow users to see in the sub-micron scale. Using the new pseudo-super resolution capability of the Zeiss Airyscan, we imagined tdTom-astros post passive clarity in optical plates. We resolved minor processes at near super resolution (Fig 4A and 4B; S4 Movie). Some processes remained fragmented post computational analyses due to the limitations of tdTomato diffusion in the minor astroglial processes. However, most reporters or immunostaining cannot distinguish very fine, peri-synaptic processes of astroglia by light microscopic methods (typically imaged with electron microscopy). Rather, the CLARITY method used here allows for regional 3D reconstruction and analytics of astroglia. Also, at this level of resolution users can easily identify biological targets of interest such as synaptic markers or protein trafficking events in thick tissue within minor astroglia processes to further elucidate astroglia biology.

## Discussion

Astroglia are arguably the most diverse CNS cell type. They hold essential roles in almost every part of the CNS homeostasis[15]. When astrocytes are ablated, neurons die[16]. Not surprisingly, astrocyte dysfunction induces a cascade of detrimental effects to the CNS and non-CNS, such as the GI tract[8, 10]. Fortunately, over the years we have identified some key events that exacerbate certain disorders, such as the downregulation of potassium channel Kir4.1, or glutamate transporter, Glt1, in Huntington’s disease and amyloid lateral sclerosis[8, 17]. This abnormal phenomenon renders neurons susceptible to hyperexcitability, toxicity, and eventually death[8]. To overcome this abnormal chain of events, groups are attempting to restore astrocytic function with adeno-associated viruses that contain an astrocyte specific serotropism[8]. In addition, there has been a recent push for glia progenitor transplantations in these models in hopes that progenitors without disease causing mutations will give rise to mature, healthy astroglia in the CNS to maintain homeostasis[18].

One major challenge over the past decade has been our ability to accurate image in high detail entire astrocyte populations. Murine astroglia can be much larger than 100uM in thickness with thousands of elaborate processes that interact with over 100k synapses[5]. Prior methodologies required sectioning of tissue, a method prone to high error due to its requirement of extensive human handling, cryo-freezing, and an irreversible mounting step. Finally, due to the thickness of astroglia, it is extremely challenging to obtain an entire astrocyte in one section. In comparison, CLARITY and other tissue clearing techniques allow users to take thick intact tissue and reconstruct such tissue with multiple different microscope systems[12]. Lastly, users of CLARITY are able to remove prior antibodies, re-stain with new antibodies, and re-image the same cells with another batch of biological targets. These reasons make CLARITY an ideal platform for imaging astroglia[12].

To remove the need for light sheet microscopy, we performed serial sectioned whole brain into 500um– 1mm sections using commercially available tissue matrixes. Next, we placed sequential sections into corresponding wells of optical well plates. The tissue sections were then never removed from the optical well plates and all procedures were performed in the optical wells, limiting human-handling. At the end of the CLARITY procedure we placed optical plates into a plate reader onto both single and multi-photon microscopes for imaging. We performed Z-stack tiling of all thick coronal brain slices. Following imaging, all files were imported to Bitplane Imaris and subjected to automated morphological reconstruction and cell count. On a side note, images before and after analyses are capable of being stacked onto other images to allow users to generate a reconstructed tissue, which is made easier by using corresponding optical wells. Tissue sections not only make it easy for basic science labs to perform this procedure but also dramatically reduces the image file size allowing users without top tier computers to be able to 3D render and process the imaging files.

We have established an alternative approach to CLARITY that allows basic science labs to perform this methodology. It dramatically reduces file size, imaging requirements, human handling of samples and allows users to perform automated 3D cellular reconstruction and analytics. Furthermore, for the first time we used CLARITY to morphologically reconstruct astroglial minor and major processes at pseudo-super resolution. Taken this tool as a means to pursue other astroglia work will be extremely beneficial. To date, astroglia are still very misunderstood, but allowing scientists to look over vast tissue at astroglia in super resolution with the ability to re-stain will likely yield groundbreaking findings in both health and disease.

## Supporting Information

S1 Fig a) tdTom-astros that co-localized with Glt1-eGFP astrocytes were manually counted with Imaris software and automatically counted using Bitplane Imaris’s spot detection.
(TIF)

S1 Movie 1000uM depth of the motor cortex from the BAC-GLT1-eGFP/tdTom-astros mouse. (MOV)

S2 Movie Morphological reconstruction of tdTom-astros astroglia-astroglia interactions. (MOV)

S3 Movie Morphological reconstruction of tdTom-astros shows cellular size heterogeneity. (MOV)

S4 Movie Minor and major processes of tdTom-astros in super resolution. (MOV)

## References

[1] Zhang Y, Barres BA. Astrocyte heterogeneity: an underappreciated topic in neurobiology. Curr Opin Neurobiol. 2010;20(5):588–94. 10.1016/j.conb.2010.06.005 . 20655735

[2] Oberheim NA, Goldman SA, Nedergaard M. Heterogeneity of astrocytic form and function. Methods Mol Biol. 2012;814:23–45. 10.1007/978-1-61779-452-0_3 22144298 PMC3506190

[3] Benarroch EE. Neuron-Astrocyte Interactions: Partnership for Normal Function and Disease in the Central Nervous System. Mayo Clinic Proceedings. 2005;80(10):1326–38. Available: 10.4065/80.10.1326. 16212146

[4] Volterra A, Meldolesi J. Astrocytes, from brain glue to communication elements: the revolution continues. Nat Rev Neurosci. 2005;6(8):626–40. 10.1038/nrn1722 . 16025096

[5] Halassa MM, Fellin T, Takano H, Dong JH, Haydon PG. Synaptic islands defined by the territory of a single astrocyte. J Neurosci. 2007;27(24):6473–7. 10.1523/JNEUROSCI.1419-07.2007 . 17567808 PMC6672436

[6] Kaiser M, Maletzki I, Hulsmann S, Holtmann B, Schulz-Schaeffer W, Kirchhoff F, et al Progressive loss of a glial potassium channel (KCNJ10) in the spinal cord of the SOD1 (G93A) transgenic mouse model of amyotrophic lateral sclerosis. J Neurochem. 2006;99(3):900–12. 10.1111/j.1471-4159.2006.04131.x . 16925593

[7] Olsen ML, Campbell SC, McFerrin MB, Floyd CL, Sontheimer H. Spinal cord injury causes a wide-spread, persistent loss of Kir4.1 and glutamate transporter 1: benefit of 17 beta-oestradiol treatment. Brain. 2010;133(Pt 4):1013–25. 10.1093/brain/awq049 20375134 PMC2850584

[8] Tong X, Ao Y, Faas GC, Nwaobi SE, Xu J, Haustein MD, et al Astrocyte Kir4.1 ion channel deficits contribute to neuronal dysfunction in Huntington's disease model mice. Nat Neurosci. 2014;17(5):694–703. 10.1038/nn.3691 24686787 PMC4064471

[9] Basso M, Pozzi S, Tortarolo M, Fiordaliso F, Bisighini C, Pasetto L, et al Mutant copper-zinc superoxide dismutase (SOD1) induces protein secretion pathway alterations and exosome release in astrocytes: implications for disease spreading and motor neuron pathology in amyotrophic lateral sclerosis. J Biol Chem. 2013;288(22):15699–711. 10.1074/jbc.M112.425066 23592792 PMC3668729

[10] Cabarrocas J, Savidge TC, Liblau RS. Role of enteric glial cells in inflammatory bowel disease. Glia. 2003;41(1):81–93. 10.1002/glia.10169 . 12465048

[11] Helmchen F, Denk W. Deep tissue two-photon microscopy. Nat Methods. 2005;2(12):932–40. 10.1038/nmeth818 . 16299478

[12] Yang B, Treweek JB, Kulkarni RP, Deverman BE, Chen CK, Lubeck E, et al Single-cell phenotyping within transparent intact tissue through whole-body clearing. Cell. 2014;158(4):945–58. 10.1016/j.cell.2014.07.017 25088144 PMC4153367

[13] Yang Y, Vidensky S, Jin L, Jie C, Lorenzini I, Frankl M, et al Molecular comparison of GLT1+ and ALDH1L1+ astrocytes in vivo in astroglial reporter mice. Glia. 2011;59(2):200–7. Epub 2010/11/04. 10.1002/glia.21089 21046559 PMC3199134

[14] Herculano-Houzel S. The glia/neuron ratio: how it varies uniformly across brain structures and species and what that means for brain physiology and evolution. Glia. 2014;62(9):1377–91. 10.1002/glia.22683 . 24807023

[15] Garcia-Marques J, Lopez-Mascaraque L. Clonal identity determines astrocyte cortical heterogeneity. Cereb Cortex. 2013;23(6):1463–72. 10.1093/cercor/bhs134 . 22617854

[16] Tsai HH, Li H, Fuentealba LC, Molofsky AV, Taveira-Marques R, Zhuang H, et al Regional astrocyte allocation regulates CNS synaptogenesis and repair. Science. 2012;337(6092):358–62. 10.1126/science.1222381 22745251 PMC4059181

[17] Guo Y, Duan W, Li Z, Huang J, Yin Y, Zhang K, et al Decreased GLT-1 and increased SOD1 and HO-1 expression in astrocytes contribute to lumbar spinal cord vulnerability of SOD1-G93A transgenic mice. FEBS Lett. 2010;584(8):1615–22. 10.1016/j.febslet.2010.03.025 . 20303959

[18] Haidet-Phillips AM, Maragakis NJ. Neural and glial progenitor transplantation as a neuroprotective strategy for Amyotrophic Lateral Sclerosis (ALS). Brain Res. 2015;1628(Pt B):343–50. 10.1016/j.brainres.2015.06.035 . 26187754 PMC9152639

