# Supplementary figures and images for: Astroglia in Thick Tissue with Super Resolution and Cellular Reconstruction
Source: PLoS One. 2016 Aug 5;11(8):e0160391. doi: 10.1371/journal.pone.0160391 (PMC4975496; doi:10.1371/journal.pone.0160391)

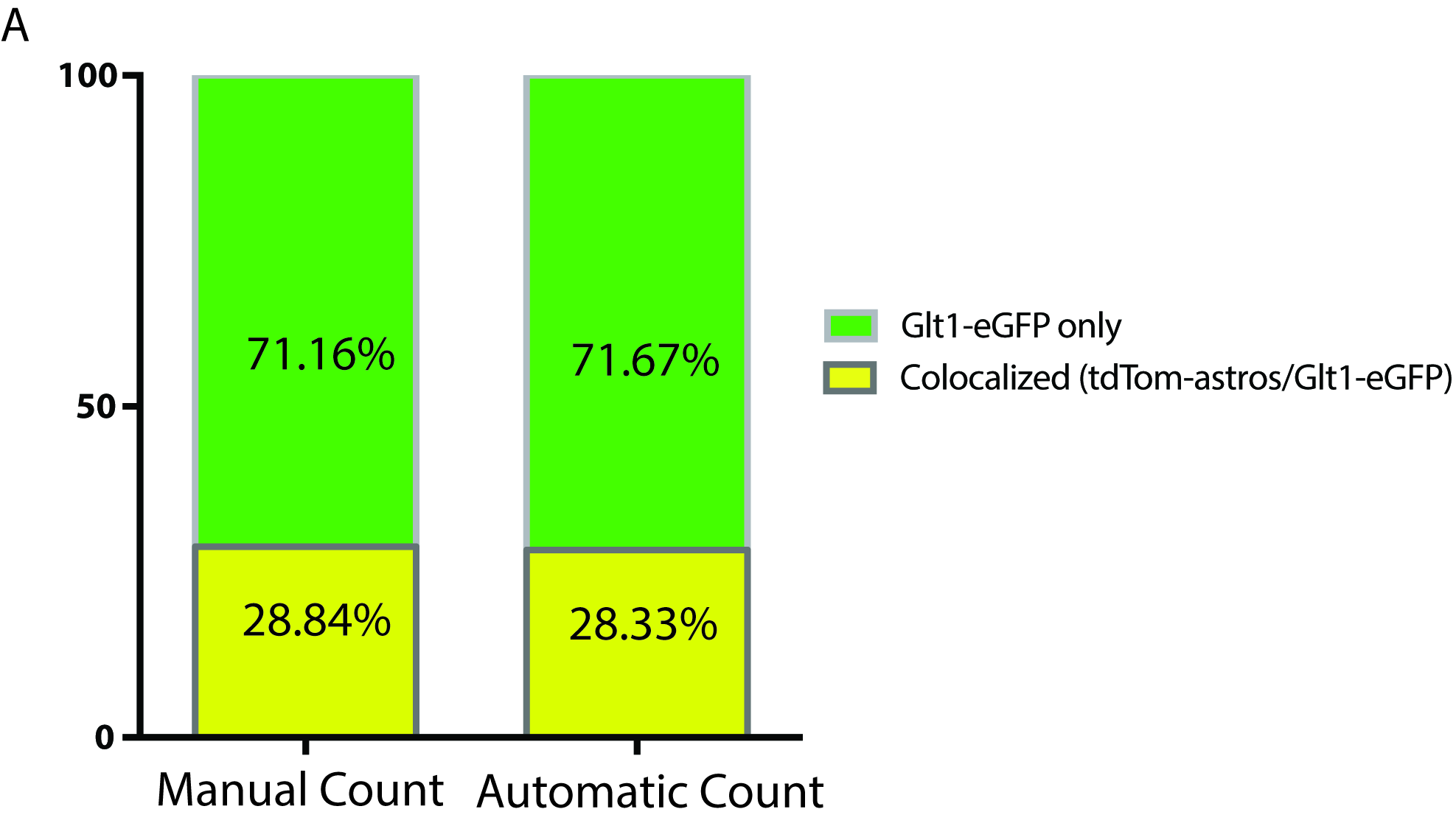

Supplement: S1 Fig — a) tdTom-astros that co-localized with Glt1-eGFP astrocytes were manually counted with Imaris software and automatically counted using Bitplane Imaris’s spot detection. (TIF) [file pone.0160391.s001.tif]
